# Herbal Formula AC591 Protects Against Cisplatin‐Induced Hearing Loss by Inhibiting p53‐Mediated Ferroptosis

**DOI:** 10.1002/smmd.70054

**Published:** 2026-09-04

**Authors:** Yunhao Wu, Yu Tao, Lin Wei, Qiuping Liu, Jiaxin Liu, Wen Jiang, Weiwei Shi, Peng Cao, Xiaolong Fu, Cheng Cheng

**Affiliations:** ^1^ Shandong Provincial Hospital, Medical Science and Technology Innovation Center, School of Clinical and Basic Medical Sciences Shandong First Medical University and Shandong Academy of Medical Sciences Jinan China; ^2^ School of Pharmacy Bengbu Medical University Bengbu China; ^3^ Department of Otolaryngology‐Head and Neck Surgery Affiliated Hospital of Jiangnan University Wuxi China; ^4^ Department of Otorhinolaryngology Qilu Hospital of Shandong University Jinan China; ^5^ Department of Otolaryngology The First Affiliated Hospital of Zhejiang Chinese Medical University Hangzhou China; ^6^ School of Pharmacy Nanjing University of Chinese Medicine Nanjing China

**Keywords:** 18β‐glycyrrhetinic acid, AC591, cisplatin, hearing loss, p53

## Abstract

Cisplatin is a widely utilized chemotherapeutic agent for the treatment of malignant cancers. However, its clinical application is significantly restricted due to its severe ototoxicity. Therefore, identifying protective medications to prevent cisplatin‐induced hearing loss (CIHL) is of paramount importance. Here, we show that AC591, derived from the classical herbal formula “Huangqi Guizhi Wuwu decoction,” exhibits notable protective effects on cisplatin‐induced ototoxicity. Our findings indicate that pretreatment with AC591 mitigates cisplatin‐induced auditory dysfunction, with its mechanism of action linked to the inhibition of p53‐mediated ferroptosis. By identifying the active components of AC591 in serum and cochlear tissues following oral administration, we recognized 18β‐glycyrrhetinic acid as an effective constituent for hearing protection. Furthermore, we confirmed that 18β‐glycyrrhetinic acid exerted otoprotective effects by binding to p53 and regulating proteins associated with ferroptosis. Collectively, our findings suggest that AC591 and 18β‐glycyrrhetinic acid may serve as promising agents for preventing CIHL.

## Key Points

- AC591 exhibits a significant protective effect against cisplatin‐induced ototoxicity.
- The attenuation of cisplatin‐induced hearing impairment by AC591 occurs primarily through the p53‐mediated ferroptosis pathway.
- 18β‐glycyrrhetinic acid is identified as the principal compound in AC591 that is responsible for its protective effects on cisplatin‐induced hearing loss.

## Graphical abstract

AC591 and its core active component 18β‐glycyrrhetinic acid effectively alleviate cisplatin‐induced ototoxicity by binding p53 and suppressing p53‐mediated ferroptosis, representing promising agents for preventing cisplatin‐related hearing loss.

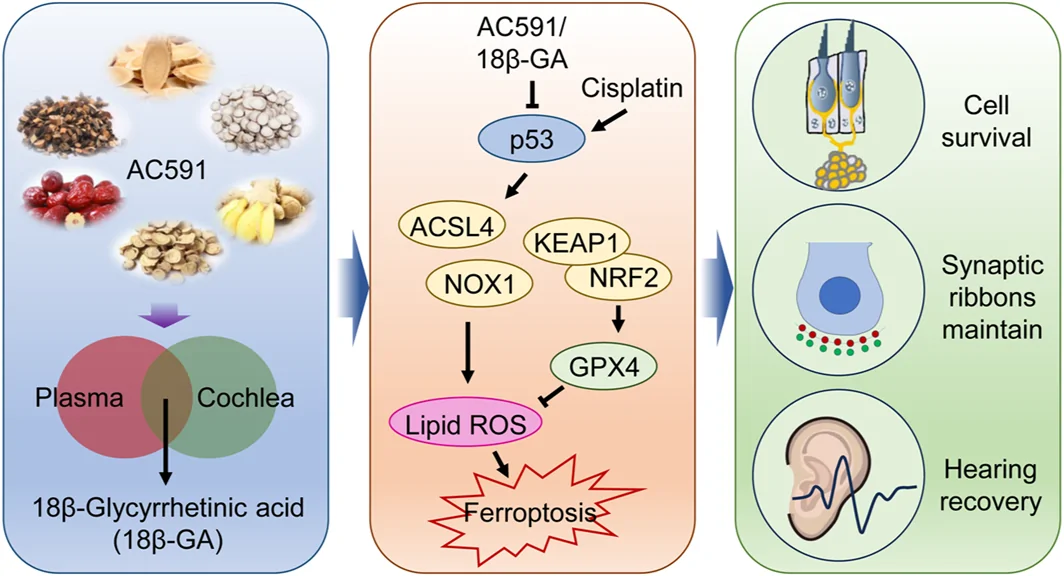

## 1 Introduction

Sensorineural hearing loss (SNHL) is the most prevalent form of sensorineural deficit, affecting individuals worldwide. This condition arises from various factors, including ototoxic drugs, noise exposure, aging, and infections. SNHL is characterized by the impairment of mechanosensory hair cells and spiral ganglion neurons (SGNs), along with the disruption of ribbon synapses that facilitate their communication [1, 2, 3]. Cisplatin chemotherapy is an effective treatment for several human cancers, including lung, head, neck, bladder, and testicular cancers [4]. However, it is associated with the risk of severe and irreversible deafness. Studies indicate that the ototoxicity of cisplatin may result from oxidative stress imbalance, apoptosis, or ferroptosis in cochlear cells [5, 6, 7]. Currently, cisplatin‐induced SNHL is permanent and there are no effective pharmacological interventions available to mitigate its ototoxic effects.

AC591 is a standardized extract derived from Huangqi Guizhi Wuwu decoction (HGWD), which comprises Astragali Radix, Paeonia Radix Alba, Cinnamomi Ramulus, Zingiberis Rhizoma, Jujubae Fructus, and Glycyrrhiza. HGWD is a traditional Chinese medicine decoction first documented in the “Synopsis of the Golden Chamber” during the Eastern Han Dynasty, primarily used for treating blood‐arthralgia, numbness, and rheumatoid arthritis [8]. Research has demonstrated that HGWD possesses anti‐inflammatory, antioxidative, anti‐tumor, and neuroprotective properties [9, 10, 11, 12, 13]. Furthermore, HGWD has been shown to mitigate the toxicity of the platinum‐based drug oxaliplatin and improve symptoms associated with peripheral neuropathy in clinical studies [14, 15]. As a standardized extract of HGWD, AC591 has been proven to prevent oxaliplatin‐induced neuropathy and may serve as a potential adjuvant for managing sensory‐related symptoms in clinical practice [16]. However, it remains unclear whether AC591 can provide protection against cisplatin‐induced hearing loss (CIHL).

In this study, we assessed the protective effect of AC591 on CIHL and investigated the underlying mechanisms responsible for this effect. Additionally, we identified the potential active constituent of AC591 that contributes to hearing protection and validated the mechanisms of action involved.

## 2 Materials and Methods

### 2.1 Preparation of AC591

AC591 was provided by Nanjing University of Chinese Medicine (Nanjing, China). The preparation process of AC591 has been detailed in previous studies [16]. In brief, after a 30‐min maceration, a mixture of Astragali Radix, Paeonia Radix Alba, Cinnamomi Ramulus, Zingiberis Rhizoma, Jujubae Fructus, and Glycyrrhiza was subjected to reflux and subsequently filtered. This refluxing process was performed twice after which the combined filtrates were desiccated and filtered through a 0.2 μm PTFE syringe filter.

### 2.2 Animal Experiments

Male C57BL/6 mice (6 weeks old) were obtained from Gempharmatech Co. Ltd. (Nanjing, China). The mice were administered AC591 at three different doses (2.34, 4.68, and 9.36 g/kg) via intragastric administration over a period of 3 weeks. At the last week of this period, the mice were injected intraperitoneally with cisplatin (4 mg/kg) for one consecutive week to establish an ototoxicity model. Two days post‐cisplatin treatment, the mice were anesthetized with pentobarbital (1%) via intraperitoneal injection and their hearing function was subsequently assessed. All animal experiments received approval from the Institutional Animal Care and Use Committee of Shandong First Medical University and complied with the National Institutes of Health Guide for the Care and Use of Laboratory Animals.

Cisplatin preparation: The cisplatin reagent used in this study was a cisplatin lyophilized powder (Solarbio, IC0440). Prior to each experiment, cisplatin powder was freshly dissolved in sterile 0.9% sodium chloride solution to a working concentration of 0.4 mg/mL. The injection volume was adjusted individually according to the body weight of each mouse to ensure a final dose of 4 mg/kg via intraperitoneal administration.

### 2.3 Auditory Brainstem Response (ABR) Audiometry

ABR measurements were conducted as previously described [17]. Briefly, after anesthetizing the mice with pentobarbital sodium (100 mg/kg), they were placed on a warming pad maintained at 37°C. Needle electrodes were positioned at the vertex of the skull and beneath the ears. ABR thresholds were recorded at frequencies of 4, 8, 12, 16, 24, and 32 kHz using a Tucker–Davis Technologies (TDT) System.

### 2.4 Distortion Product Otoacoustic Emission (DPOAE) Test

DPOAEs were recorded using TDT hardware and software, with a modified pipette tip inserted into the external ear canal of the mouse. The TDT Real‐Time Signal Processing System II measured the distortion product at 2*ƒ*1 − *ƒ*2 (where *ƒ*2/*ƒ*1 = 1.22, and *ƒ*2 was 5 dB lower than *ƒ*1) at frequencies of 8, 12, 16, 24, and 32 kHz. F2 levels were swept from 80 to 10 dB SPL; the DPOAE threshold was defined as 2*ƒ*1 − *ƒ*2 exceeding the noise floor by ≥ 6 dB, with *f*2 levels inducing hearing thresholds at 5 dB SPL.

### 2.5 Immunostaining Analysis

Cochlear tissues were fixed in 4% paraformaldehyde and subsequently decalcified with 0.5 M EDTA. The following day, cochleae were sectioned, permeabilized, and blocked with 10% donkey serum for 1.5 h, followed by incubation with the corresponding primary antibodies: anti‐Myosin 7a (Abcam, MA, USA), anti‐NF200 (Abcam, MA, USA), anti‐Ctbp2 (Abcam, MA, USA), anti‐GluR2 (Merck Millipore, Darmstadt, Germany), anti‐p53 (Abcam, MA, USA), anti‐4‐HNE (Thermo Fisher, MA, USA), and anti‐3‐NT (Abcam, MA, USA) at 4°C overnight. Cochlear samples were washed three times with PBS and incubated with the appropriate secondary antibodies (Thermo Fisher, Waltham, USA) for 1.5 h at room temperature. After washing with PBS for three times, the samples were visualized using a confocal fluorescence microscope (Zeiss, Germany).

### 2.6 RNA Sequencing

Total RNA from the cochlea was isolated using the FastPure Cell/Tissue Total RNA Isolation Kit V2 (Vazyme, Nanjing, China). The integrity of the RNA was assessed using the Nano 6000 Assay Kit on a Bioanalyzer 2100 system (Agilent Technologies, CA, USA). Library construction was performed using the TruSeq Stranded mRNA LT Sample Prep Kit (Illumina, CA, USA). The library was quantified using a Qubit 2.0 fluorometer, diluted to 1.5 ng/μL, and the insert size was determined on the Bioanalyzer 2100. Sequencing of the library was conducted on an Illumina NovaSeq 6000. The enrichment of differentially expressed genes in KEGG pathways was analyzed using the clusterProfiler R package.

### 2.7 Quantitative PCR

After extraction from the cochlea, RNA was reverse transcribed into cDNA using the HiScript III first Strand cDNA Synthesis Kit (Vazyme, Nanjing, China). Quantitative PCR (qPCR) was performed with the AceQ universal SYBR qPCR Master Mix (Vazyme, Nanjing, China) on a CFX96 real‐time PCR system (Bio‐Rad, CA, USA). GAPDH served as the control in the same samples, and results were calculated using the comparative cycle threshold (ΔΔCt) method. The primers used for quantitative PCR were as follows: *ACSL4*: CTCACCATTATATTGCTGCCTGT (Forward sequence 5′‐3′), TCTCTTTGCCATAGCGTTTTTCT (Reverse sequence 5′‐3′); *NOX1*: GGTTGGGGCTGAACATTTTTC (Forward sequence 5′‐3′), TCGACACACAGGAATCAGGAT (Reverse sequence 5′‐3′); *PTGS2*: TTCAACACACTCTATCACTGGC (Forward sequence 5′‐3′), AGAAGCGTTTGCGGTACTCAT (Reverse sequence 5′‐3′); *NRF2*: TCTTGGAGTAAGTCGAGAAGTGT (Forward sequence 5′‐3′), GTTGAAACTGAGCGAAAAAGGC (Reverse sequence 5′‐3′); *GPX4*: GATGGAGCCCATTCCTGAACC (Forward sequence 5′‐3′), CCCTGTACTTATCCAGGCAGA (Reverse sequence 5′‐3′).

### 2.8 Lipid Peroxidation Assay

#### 2.8.1 For Cochlear Tissue Samples

After the mice were sacrificed, mouse cochleae were rapidly dissected on ice in pre‐chilled sterile PBS. The bony capsule, vestibular structures and stria vascularis were carefully micro‐dissected to isolate intact basilar membranes. Freshly prepared basilar membrane samples were incubated with 10 μM C11 BODIPY 581/591 probe diluted in pre‐warmed DMEM/F12 medium for 30 min at 37°C in the dark. After incubation, tissues were rinsed three times with ice‐cold PBS (5 min per wash) to remove residual unbound probe, flat‐mounted with anti‐fade mounting medium, and immediately imaged via confocal fluorescence microscopy. All steps were performed strictly protected from light to prevent fluorophore quenching and ex vivo artifactual lipid peroxidation.

#### 2.8.2 For Cultured HEI‐OC1 Cells

Cells were plated on confocal culture dishes and received the designated experimental treatments. After aspiration of the culture medium, cells were gently washed twice with pre‐warmed PBS. The C11 BODIPY 581/591 probe was diluted to 10 μM in serum‐free, phenol red‐free DMEM, and then added to cells for 30 min incubation at 37°C in a humidified 5% CO_2_ incubator in the dark. Following incubation, the probe solution was removed, and cells were rinsed three times with pre‐warmed PBS. Fluorescence images were acquired immediately by confocal microscopy.

### 2.9 Liquid Chromatography‐High Resolution Mass Spectrometry (LC‐HRMS) Analysis

#### 2.9.1 Chromatographic and Mass Spectrometric Conditions

Chromatographic separation was performed on an ACQUITY UPLC HSS T3 column (100 mm × 2.1 mm, 1.8 μm, Waters) maintained at 45°C. The mobile phase consisted of 0.1% formic acid in water (phase A) and acetonitrile (phase B) delivered at a flow rate of 0.35 mL/min. The gradient elution program was set as follows: 0–2 min, 5% B; 2–15 min, 5%–95% B; 15–18 min, 95% B; 18–18.1 min, 95%–5% B; 18.1–22 min, 5% B for equilibration. The injection volume was 5 μL per sample. Mass spectrometric detection was carried out on a Q‐Exactive hybrid quadrupole‐Orbitrap mass spectrometer with a heated electrospray ionization source, operated in both positive and negative ionization modes. Key parameters were set as follows: spray voltage, 3.8 kV (positive) and 3.0 kV (negative); capillary temperature, 320°C; aux gas heater temperature, 350°C; sheath gas flow rate, 35 Arb; aux gas flow rate, 8 Arb. Full‐scan acquisition was performed over m/z 100–1200 at a resolution of 70,000, with data‐dependent MS/MS acquisition at 17,500 resolution and normalized collision energies of 10, 20, and 40 eV.

#### 2.9.2 Qualitative Identification Strategy

Prior to pattern recognition, raw data were processed using Progenesis QI v3.0 software (Nonlinear Dynamics, Newcastle, UK) for baseline filtering, peak detection, integration, retention time correction, peak alignment, and normalization. Qualitative compound identification was performed based on accurate mass, MS/MS fragment ions, and isotopic distribution by matching with the TCM database. Specifically, the identity of 18β‐GA was confirmed by comparison with an authentic reference standard (purity ≥ 98%, CAS No. 471‐53‐4):

— Retention time: 11.53 min (positive mode), 11.54 min (negative mode)
— Precursor ions: m/z 471.3470 [M + H]^+^, m/z 469.3324 [M − H]^−^
— Characteristic product ions: m/z 425.34, 407.33, 355.26 (positive mode); m/z 425.34, 317.21, 299.20 (negative mode)

All spectra were fully consistent with reference spectra from the database. Quantitative analysis of 18β‐GA in serum and cochlear samples was performed using the external standard method. A calibration curve was established using serial dilutions of 18β‐GA standard solution over a linear range of 1–500 ng/mL with a correlation coefficient (*R* ^2^) > 0.99. Sample concentrations were calculated from the peak area of the target ion against the calibration curve.

### 2.10 Molecular Docking

The structure of the p53 protein was predicted using AlphaFold2. Subsequent preprocessing of this structure was carried out with the Protein Preparation Wizard in the Schrodinger software suite, which involved optimizing H‐bond assignments, minimizing energy, and eliminating water molecules. The LigPrep module in Schrodinger was utilized to process the 2D sdf structure file of compound 18β‐Glycyrrhetinic acid (18β‐GA), leading to the generation of all its 3D chiral conformations. To identify the optimal binding site, the SiteMap module in Schrodinger was employed followed by the Receptor Grid Generation module to create a fitting enclosing box that accurately encapsulated the predicted binding site. The active sites of the p53 protein were identified, and the prepared ligand, 18β‐GA, was docked at these sites using XP docking, which is recognized for its high precision. A lower docking score indicates greater binding stability of the compound to the protein.

### 2.11 Western Blot

The protein was quantified using a BCA protein assay kit obtained from Thermo (MA, USA). For each group, 40 μg of protein extract was applied to each lane and subjected to gel electrophoresis before being transferred to a PVDF membrane made by Millipore (MA, USA). Subsequently, the samples were incubated for 2 h at room temperature with a blocking solution of 5% BSA, followed by an overnight incubation at 4°C with primary antibodies. The following day, the samples underwent three washes with TBST and were then incubated with a secondary antibody conjugated to HRP. After undergoing another three washes with TBST, the bands were visualized using an ECL kit and analyzed with ImageQuant software.

### 2.12 Statistical Analysis

The data are presented as means ± standard deviation. For data analysis, GraphPad Prism 9 software was utilized. Multiple comparisons were performed using one‐way analysis of variance (ANOVA), and a *p* value of less than 0.05 was regarded as statistically significant.

## 3 Results

### 3.1 AC591 Prevents CIHL In Vivo

**FIGURE 1:**
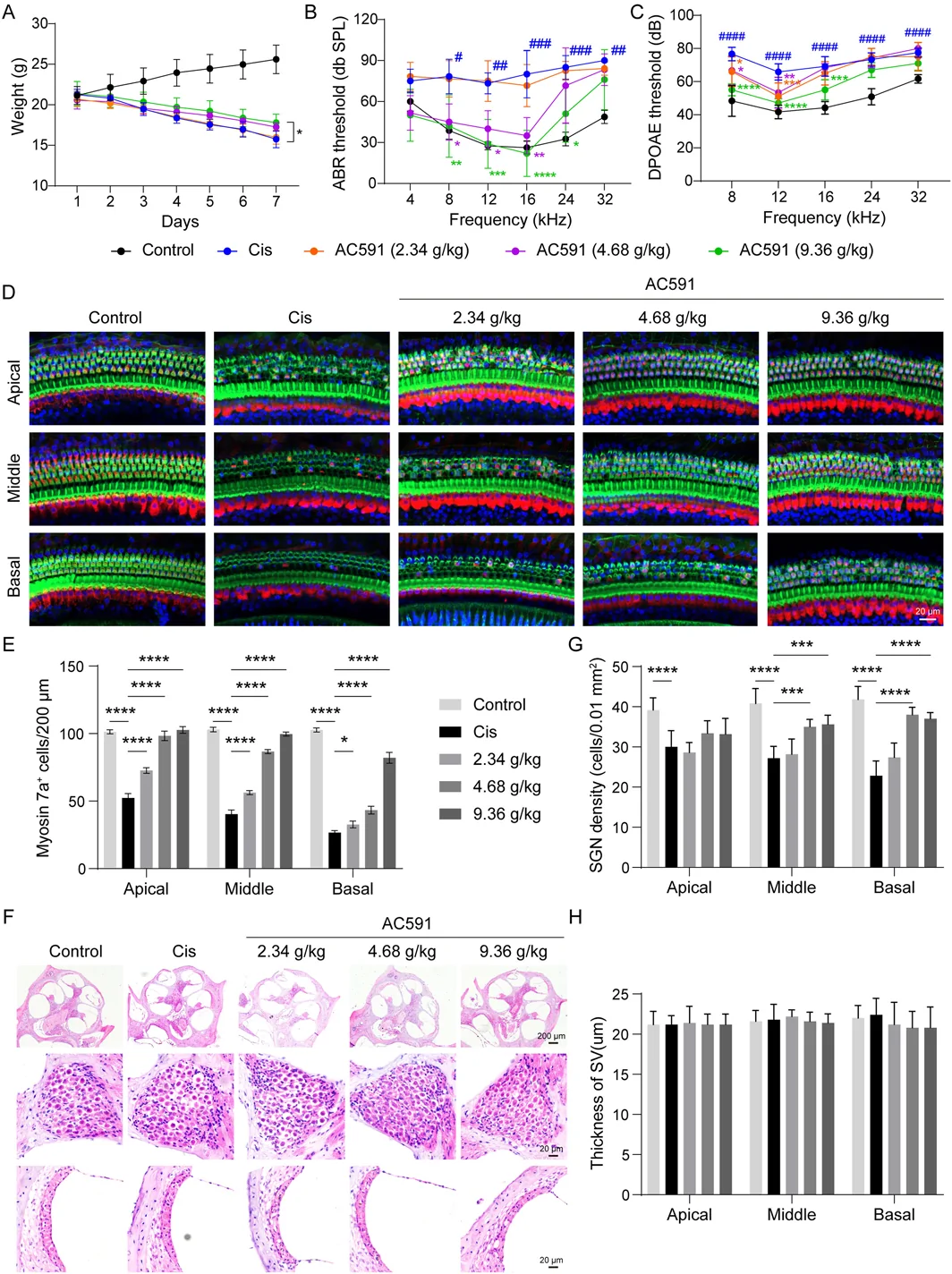
Effects of AC591 on cisplatin‐induced ototoxicity in mice. (A) Weight changes after AC591 and cisplatin treatment (*n* = 6). (B) ABR analysis of different groups (*n* = 6). (C) DPOAE analysis of different groups (*n* = 6). ^#^ *p* < 0.05, ^##^ *p* < 0.01, ^###^ *p* < 0.001, ^####^ *p* < 0.0001 versus the control group; *p < 0.05, **p < 0.01, ***p < 0.001, ****p < 0.0001 versus the cisplatin group. (D) Immunostaining of cochlear hair cells with anti‐Myosin 7a antibody and phalloidin. (E) Quantification of cochlear hair cells in the apical, middle, and basal turns in the different groups (*n* = 3). (F) HE staining of cochleae in different groups. (G) Quantification of cochlear SGNs in different groups in (F) (*n* = 5). (H) Quantification of the thickness of cochlear SV in different groups in (F) (*n* = 5). *p < 0.05, ***p < 0.001, ****p < 0.0001.

To investigate the protective effect of AC591 on hearing loss, we established a mouse model of cisplatin‐induced ototoxicity. Following intraperitoneal injection of cisplatin for 1 week, the mice exhibited significant weight loss. However, recovery was observed after pretreatment with AC591 (9.36 g/kg) (Figure 1A). ABR results demonstrated that administration of AC591 at concentrations of 4.68 and 9.36 g/kg significantly reduced cisplatin‐induced elevated thresholds at frequencies of 8, 12, 16 and 24 kHz (Figure 1B). DPOAE measurements showed that AC591 at 9.36 g/kg markedly alleviated CIHL (Figure 1C). Immunofluorescence staining of the cochlear basilar membrane revealed that AC591 protected against cisplatin‐induced cochlear hair cell loss in a dose‐dependent manner (Figure 1D,E). This finding was corroborated by immunofluorescence analysis of the frozen cochlear sections (Supporting Information S1: Figure S1A,B). In addition, hematoxylin‐eosin (HE) staining of the cochlea showed that AC591 effectively prevented the cisplatin‐induced reduction in SGNs, with no significant effect on stria vascularis thickness (Figure 1F–H). Further TUNEL staining of cochlear SGNs demonstrated that AC591 ameliorated cisplatin‐triggered SGN apoptosis (Supporting Information S1: Figure S1C,D). These results suggest that AC591 has the potential to prevent CIHL.

### 3.2 AC591 Alleviates Cisplatin‐Induced Degeneration of Cochlear Neurofilaments and Ribbon Synapses

**FIGURE 2:**
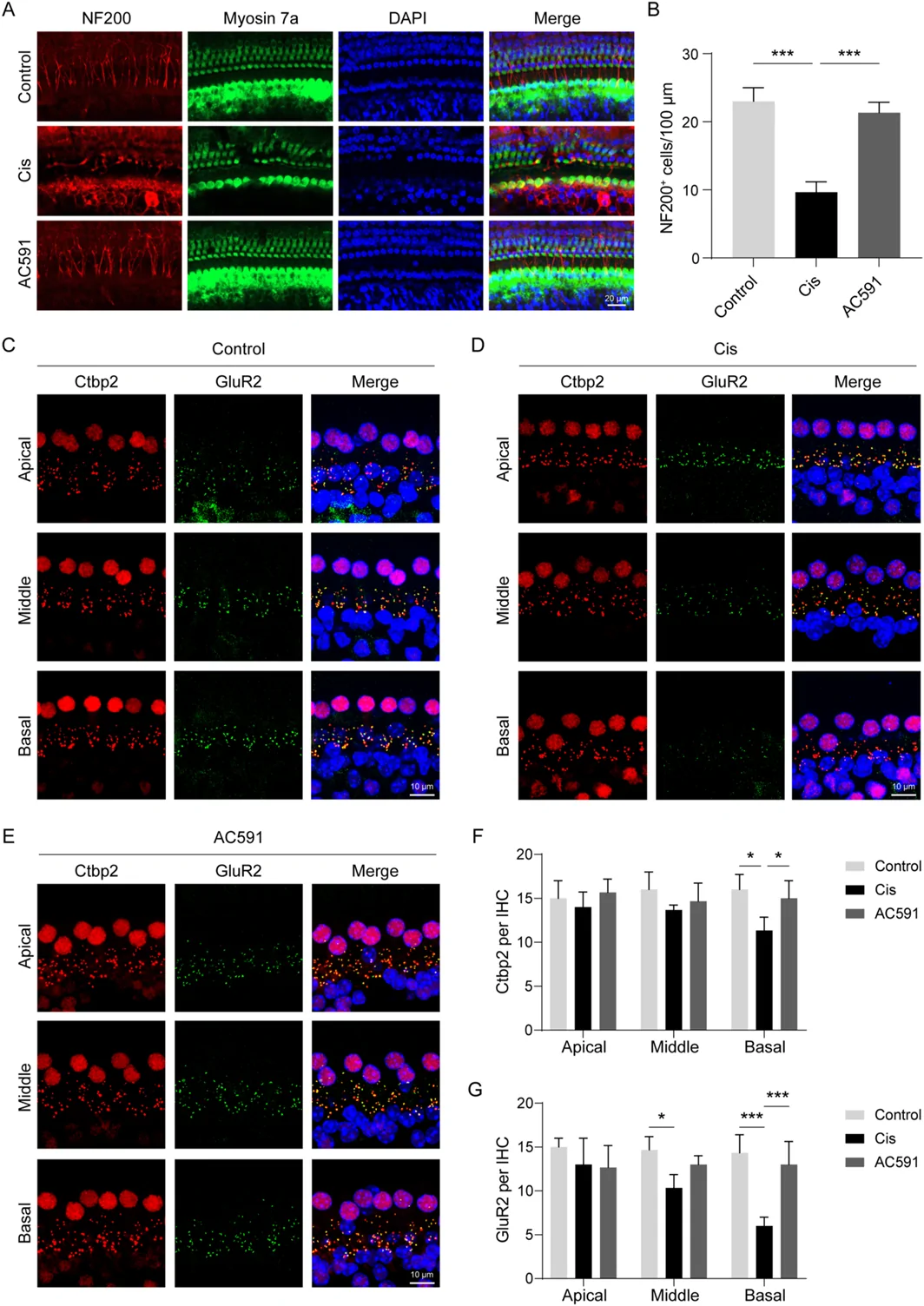
AC591 prevents cisplatin‐induced loss of cochlear neurofilaments and ribbon synapses. (A) Immunostaining of cochlear neurofilaments with anti‐NF200 antibody. (B) Quantification of NF200‐positive cells (*n* = 3). (C–E) Immunostaining of ribbon synapses with anti‐CTBP2 and anti‐GLUR2 antibodies in the apical, middle, and basal turns in the different groups. (F, G) Quantification of the number of ribbon (Ctbp2) and postsynaptic (GluR2) receptors (*n* = 3), *p < 0.05, ***p < 0.001.

The neurofilaments and ribbon synapses in the cochlea facilitate the transduction of sound signals and play a critical role in auditory function. Our findings revealed a substantial presence of auditory nerve axons connecting hair cells in the control group. However, a significant loss of axons was observed following cisplatin treatment. Notably, AC591 was effective in reversing cisplatin‐induced reduction in neurofilaments (Figure 2A,B). To investigate whether the loss of neurofilaments contributes to alterations in ribbon synapses, both ribbon and postsynaptic receptors were stained with anti‐Ctbp2 and anti‐GluR2 (Figure 2C–E) and subsequently quantified. Immunofluorescence results demonstrated that cisplatin exposure markedly diminished the number of ribbon synapses in the middle and basal turns of the cochlea, whereas AC591 treatment effectively restored the loss of ribbon synapses, as evidenced by the presynaptic dots (Figure 2F) and post‐synaptic puncta (Figure 2G) in the basal turn. Collectively, these findings indicate that AC591 restored cisplatin‐induced hearing dysfunction.

### 3.3 AC591 Regulates Cisplatin‐Activated p53 Signaling Pathway in the Cochlea

**FIGURE 3:**
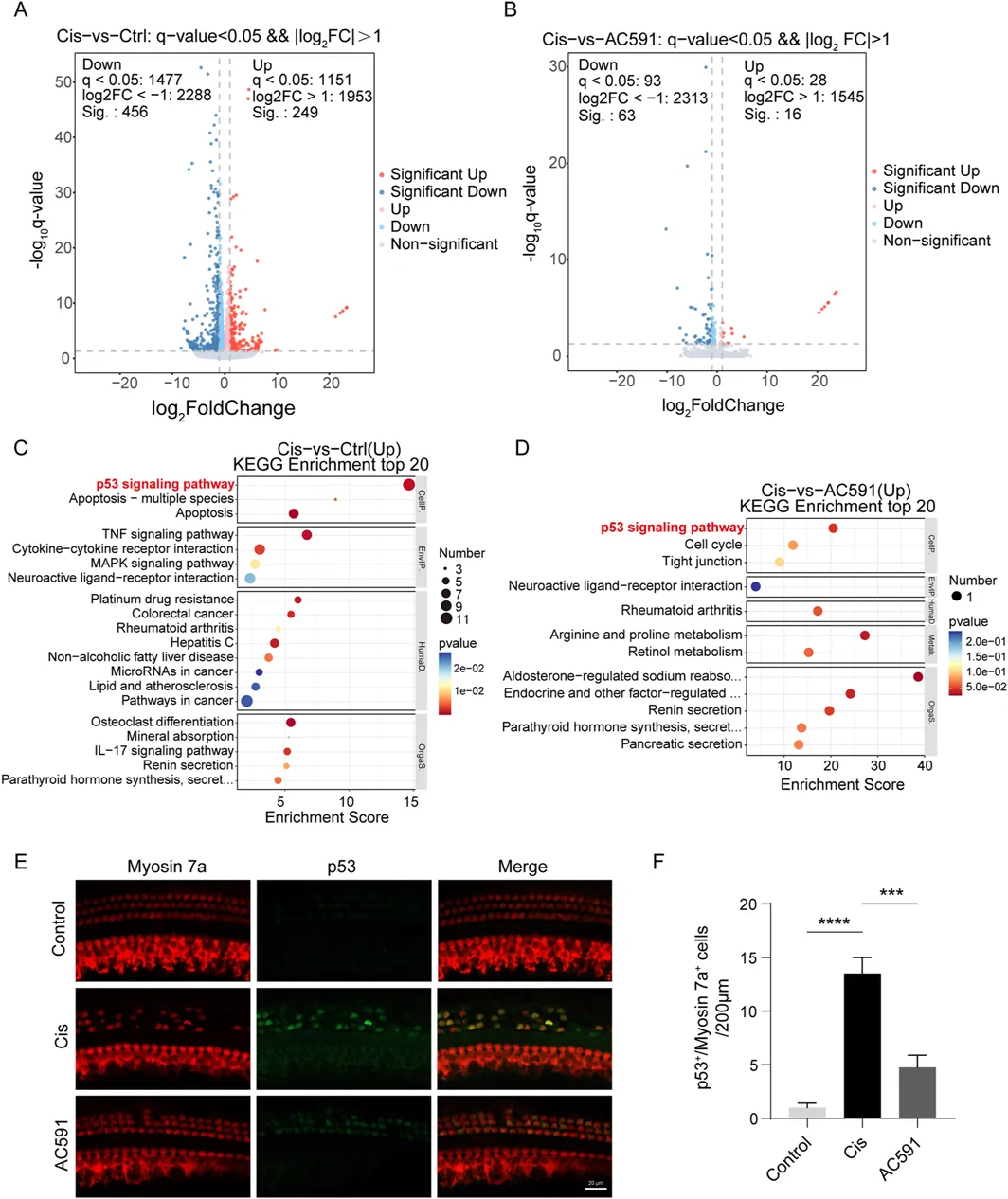
AC591 modulates cisplatin‐induced activation of the p53 signaling pathway in the mouse cochlea. (A) Volcano plot of genes with significant expression changes between the control and cisplatin groups. (B) Volcano plot of genes with significant expression changes between the cisplatin and AC591 groups. (C) KEGG enrichment analysis of the significantly changed pathways after cisplatin treatment. (D) KEGG enrichment analysis of the significantly changed pathways after AC591 administration. (E) Immunostaining of p53 in cochlear hair cells in different groups. (F) Quantification of p53‐positive hair cells in the different groups (*n* = 3), ***p < 0.001, ****p < 0.0001.

RNA sequencing (RNA‐seq) of the mouse cochlea was performed to elucidate the mechanism by which AC591 confers protection against hearing loss. The RNA‐seq analysis revealed 705 significantly altered genes, comprising 249 that were up‐regulated and 456 that were down‐regulated following cisplatin exposure compared to the control group (Figure 3A). Furthermore, 79 genes (16 up‐regulated and 63 down‐regulated) were significantly altered by AC591 treatment in comparison with the cisplatin group (Figure 3B). A Kyoto Encyclopedia of Genes and Genomes (KEGG) pathway analysis was conducted to identify the key pathways; the p53 signaling pathway was significantly activated by cisplatin treatment, whereas AC591 effectively inhibited the cisplatin‐induced activation of this pathway (Figure 3C,D). Consistent with the RNA‐seq findings, immunostaining of p53 in cochlear hair cells confirmed that AC591 reduced the elevated expression of p53 induced by cisplatin (Figure 3E,F). Collectively, these results suggest that AC591 protects against CIHL through inhibition of the p53 signaling pathway.

### 3.4 AC591 Inhibits Cisplatin‐Induced Ferroptosis in the Cochlea

**FIGURE 4:**
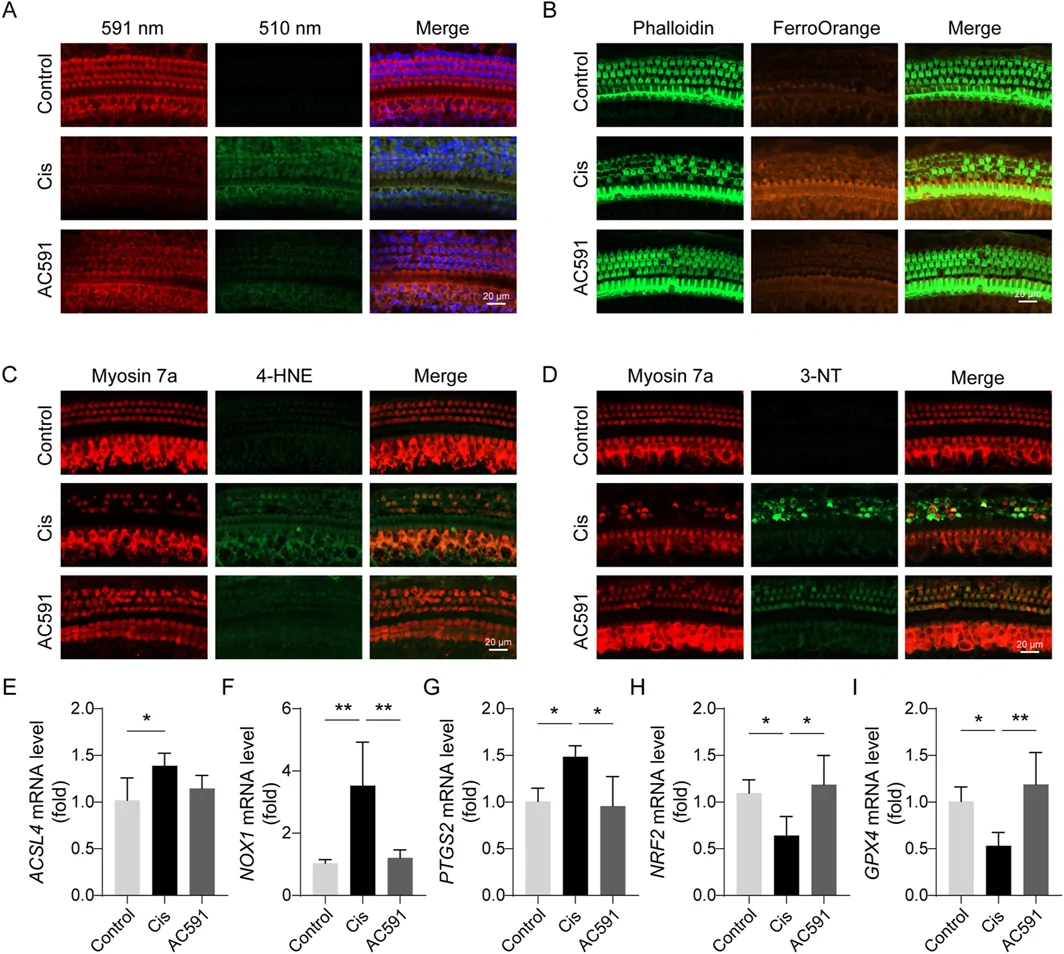
AC591 reverses cisplatin‐induced ferroptosis in the cochlea. (A) C11 BODIPY 581/591 staining for the measurement of lipid peroxidation levels. (B) FerroOrange staining for the detection of intracellular ferrous ions. (C, D) Immunostaining of 4‐HNE and 3‐NT in cochlear hair cells. (E–I) Real‐time PCR analysis of the mRNA levels of *ACSL4*, *NOX1*, *PTGS2*, *NRF2*, and *GPX4* in the mouse cochlea (*n* = 3). *p < 0.05, **p < 0.01.

Ferroptosis is implicated in cisplatin‐induced ototoxicity, with p53 reported to modulate both the canonical and non‐canonical ferroptosis pathways [7, 18]. Therefore, we aim to determine whether AC591 could mitigate cisplatin‐induced ferroptosis in cochlear hair cells. The C11 BODIPY 581/591 probe was employed to assess the lipid peroxidation levels. As illustrated in Figure 4A, AC591 significantly reduced cisplatin‐induced lipid peroxidation in hair cells. FerroOrange staining was utilized to detect intracellular ferrous ions, revealing that cisplatin treatment led to an accumulation of ferrous ions, whereas mice treated with AC591 exhibited a marked depletion of intracellular iron in cochlear hair cells (Figure 4B). Immunofluorescence analysis of the lipid peroxidation markers, particularly 4‐hydroxynonenal (4‐HNE) and 3‐nitrotyrosine (3‐NT), demonstrated that AC591 effectively decreased the elevated expression of 4‐HNE and 3‐NT triggered by cisplatin in cochlear hair cells (Figure 4C,D). In alignment with these results, qPCR analysis of mouse cochlear tissue indicated that AC591 regulated the mRNA levels of ferroptosis‐related genes including *NOX1*, *PTGS2*, *NRF2*, and *GPX4* (Figure 4E–I). Collectively, these results suggest that AC591 may ameliorate cisplatin‐induced ferroptosis in cochlear hair cells.

### 3.5 Identification of 18β‐GA as the Effective Compound of AC591 for Hearing Protection

**FIGURE 5:**
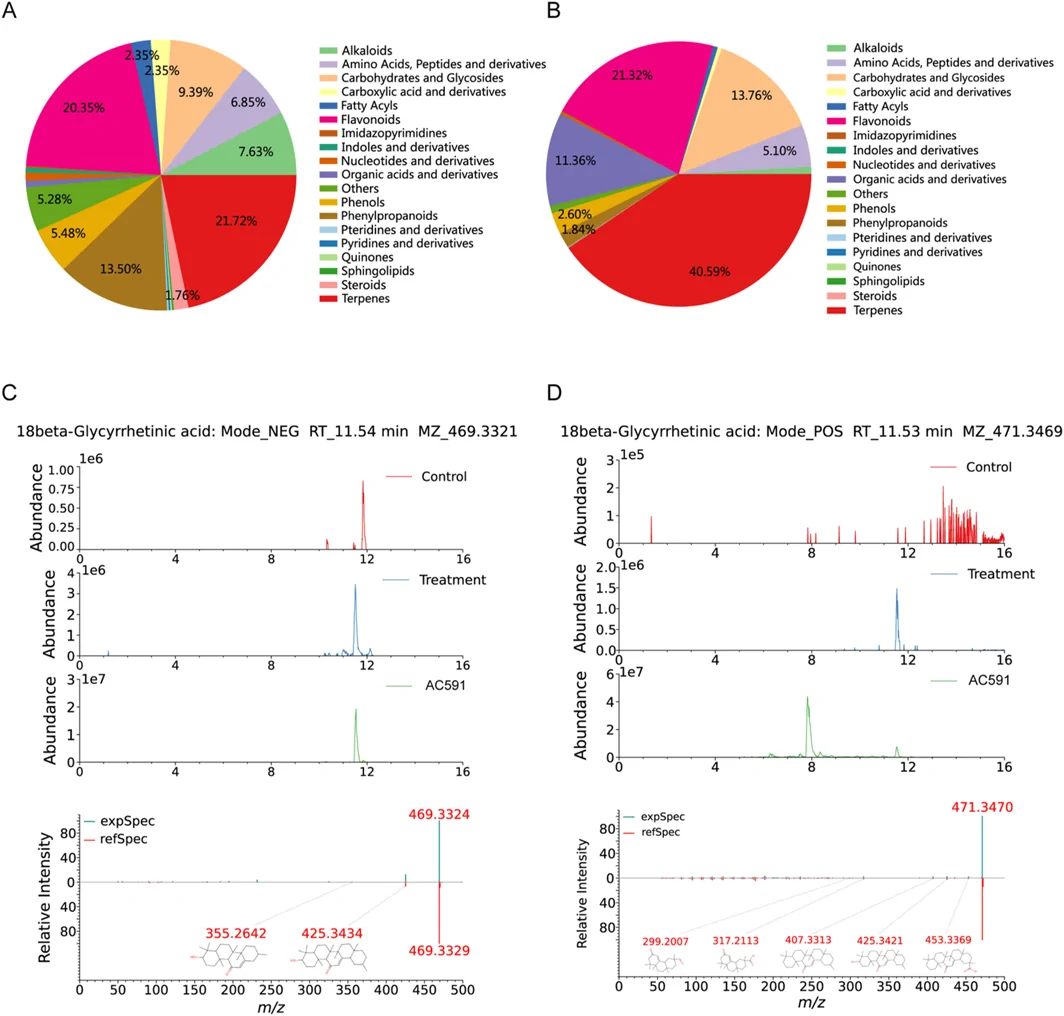
Identification of the active compound of AC591 for hearing protection. (A) Distribution diagram of the ingredients of AC591. (B) Distribution diagram of the percentage composition of the AC591 ingredients. (C) Identification of 18β‐GA in serum by mass spectrometry. (D) Identification of 18β‐GA in the cochlea by mass spectrometry.

To further elucidate the mechanism underlying the protective effect of AC591 against cisplatin‐triggered hearing loss, we conducted LC‐HRMS analysis to identify the active constituents present in serum and cochlea following oral administration of AC591. As illustrated in Figure 5A,B, AC591 is predominantly composed of terpenes, flavonoids, carbohydrates, glycosides, amino acids, peptides, and their derivatives. Following oral administration of AC591, we identified 13 compounds in serum and three compounds in the cochlea as determined by the base peak chromatogram and total ion chromatogram for serum (Supporting Information S1: Figure S2) and cochlea (Supporting Information S1: Figure S3). Notably, among the detected compounds, we observed 18β‐GA in both serum (Figure 5C) and cochlea (Figure 5D), suggesting that 18β‐GA may serve as the active ingredient in AC591 responsible for conferring hearing protection.

### 3.6 18β‐GA Binds to p53 and Protects Against Cisplatin‐Induced Ferroptosis

**FIGURE 6:**
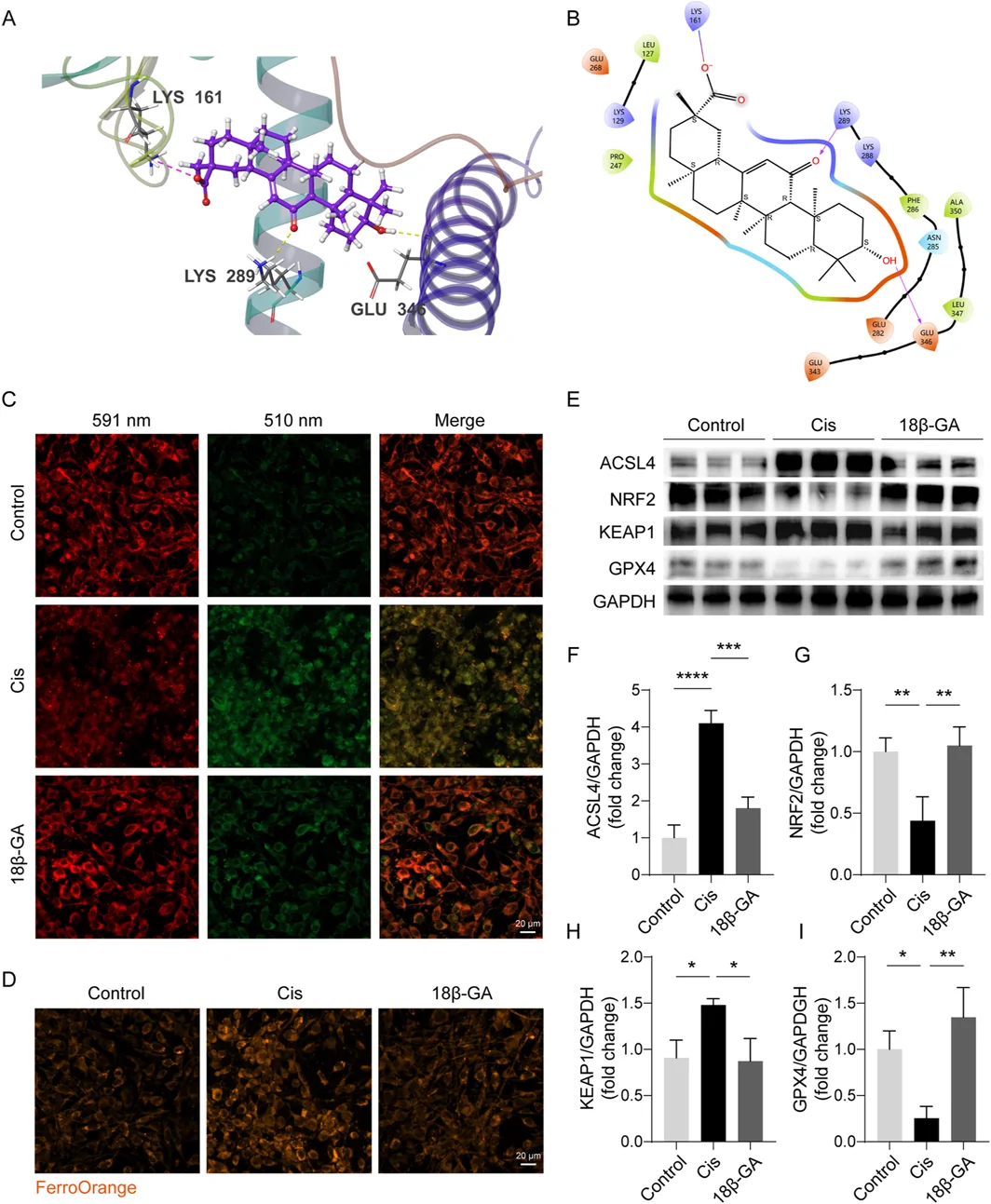
18β‐GA inhibits cisplatin‐induced ferroptosis by binding to p53. (A) The 3D binding model of 18β‐GA to p53 protein. (B) The possible binding sites of 18β‐GA and p53 in two dimensions graph using computer molecular docking analysis. (C) C11 BODIPY 581/591 staining in HEI‐OC1 cells in the different groups. (D) FerroOrange staining in HEI‐OC1 cells in the different groups. (E) WB analysis of ACSL4, NFR2, KEAP1, and GPX4 expression. (F–I) Quantification of the expression of ACSL4, NFR2, KEAP1, and GPX4 (*n* = 3), *p < 0.05, **p < 0.01, ***p < 0.001, ****p < 0.0001.

Following our findings that AC591 effectively prevented cisplatin‐induced ototoxicity by inhibiting p53‐mediated ferroptosis, we conducted a molecular docking analysis which revealed that 18β‐GA could bind to the active pocket of the p53 protein. Specifically, residue PRO247 of p53 established a hydrophobic interaction with 18β‐GA, while18β‐GA also formed a salt bridge with residue LYS161 and hydrogen bonds with residues LYS289 and GLU346 of p53 (Figure 6A,B). Additionally, staining with C11 BODIPY 581/591 and FerroOrange indicated that 18β‐GA mitigated cisplatin‐induced lipid peroxidation and decreased the accumulation of intracellular ferrous ions in House Ear Institute‐Organ of Corti 1 (HEI‐OC1) cells (Figure 6C,D). Furthermore, western blot analysis demonstrated that 18β‐GA alleviated cisplatin‐induced ferroptosis in HEI‐OC1 cells by modulating ferroptosis‐related proteins, including ACSL4, NRF2, KEAP1, and GPX4 (Figure 6E–I). Collectively, these results confirm that 18β‐GA is the primary active compound of AC591 that protects against cisplatin‐induced ototoxicity through the inhibition of p53‐mediated ferroptosis.

### 3.7 18β‐GA Protects Against Cisplatin‐Induced Hearing Impairment In Vivo

**FIGURE 7:**
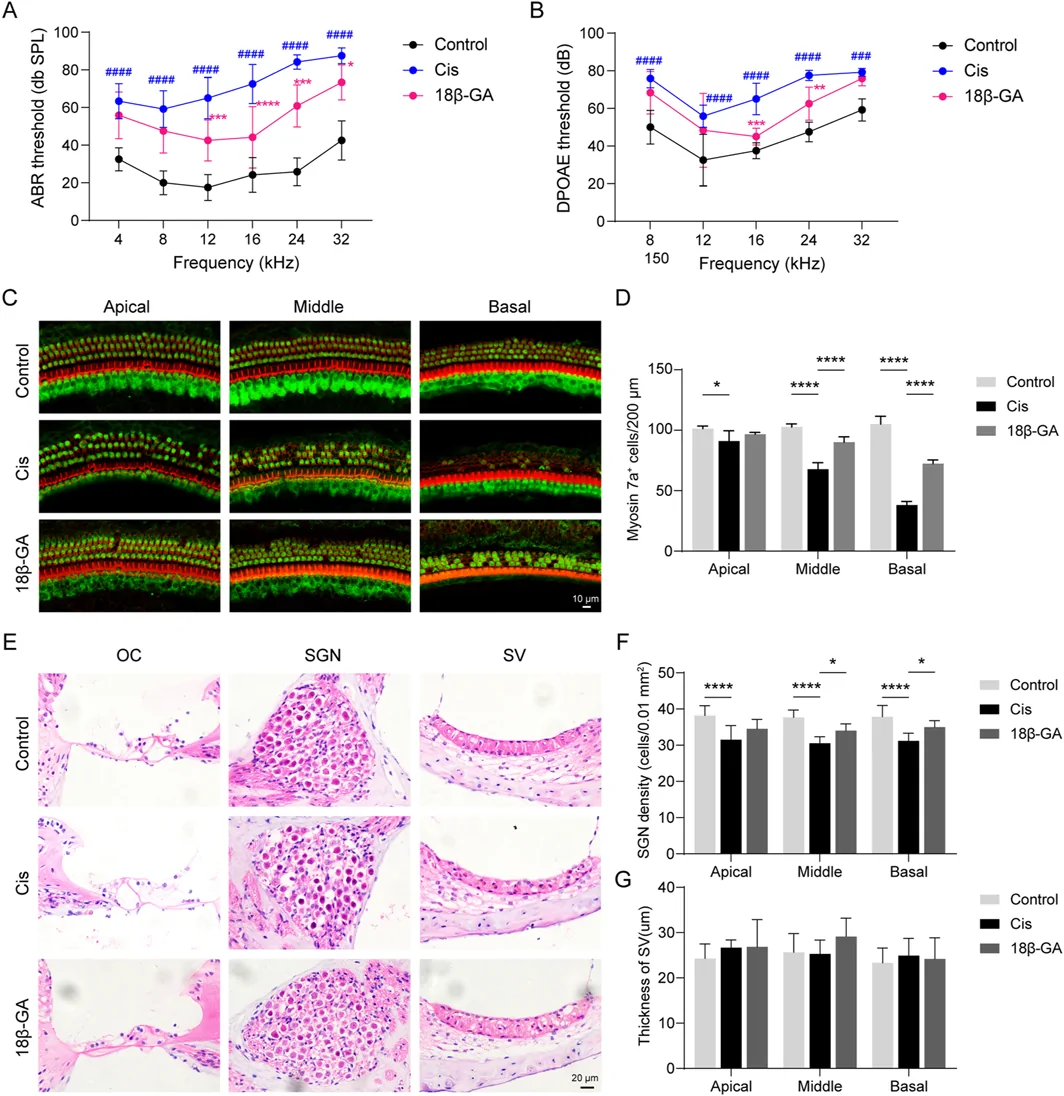
18β‐GA protects against cisplatin‐induced hearing impairment. (A) ABR analysis of different groups (*n* = 6). (B) DPOAE analysis of different groups (*n* = 6). ^###^ *p* < 0.001, ^####^ *p* < 0.0001 versus the control group; *p < 0.05, **p < 0.01, ***p < 0.001, ****p < 0.0001 versus the cisplatin group. (C) Immunostaining of cochlear hair cells with anti‐Myosin 7a antibody and phalloidin. (D) Quantification of cochlear hair cells in the apical, middle, and basal turns in the different groups (*n* = 3). (E) HE staining of cochleae in different groups. (F) Quantification of cochlear SGNs in different groups in (E) (*n* = 6). (G) Quantification of the thickness of cochlear SV in different groups in (E) (*n* = 6). *p < 0.05, ****p < 0.0001.

Next, we further validated the effects of 18β‐GA on cisplatin‐induced ototoxicity using in vivo animal experiments. Mice were intragastrically administered 18β‐GA (50 mg/kg) prior to cisplatin‐induced model establishment. Auditory functional assessments, including ABR and DPOAE measurements, revealed that 18β‐GA effectively ameliorated cisplatin‐provoked auditory dysfunction (Figure 7A,B). Immunofluorescence analysis of mouse cochlear hair cells demonstrated that 18β‐GA exerted prominent protective effects on cisplatin‐triggered hair cell loss (Figure 7C,D). In addition, HE staining of cochlear sections showed that 18β‐GA protected against cisplatin‐induced SGN loss without producing a significant impact on stria vascularis thickness (Figure 7E–G). Collectively, these findings indicate that 18β‐GA exhibits comparable pharmacological efficacy to AC591 and affords effective protection against cisplatin‐evoked hearing impairment.

### 3.8 18β‐GA Did Not Affect the Anti‐Tumor Effect of Cisplatin

**FIGURE 8:**
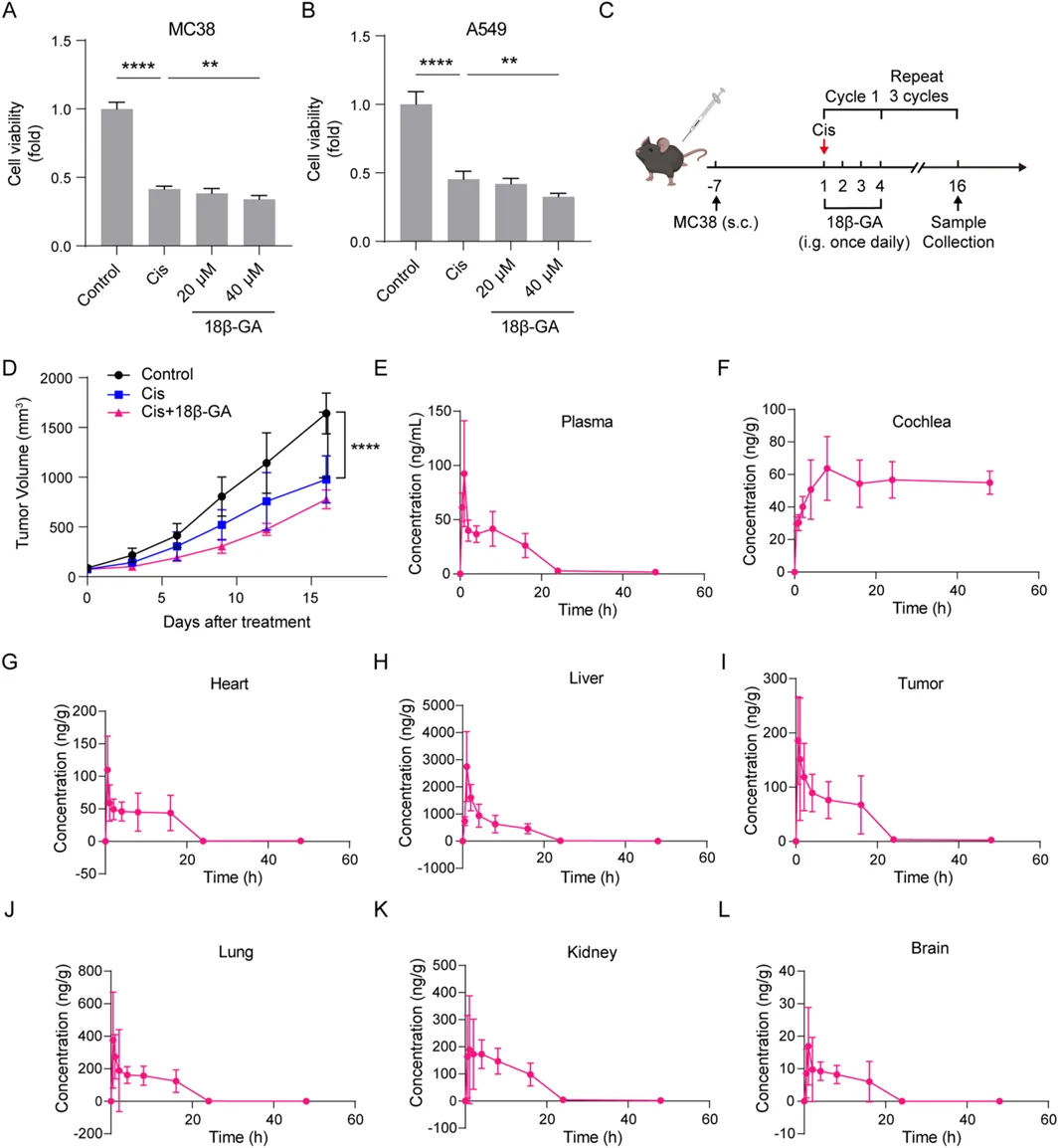
18β‐GA has no influence on the anti‐tumor activity of cisplatin. (A) Survival rate of MC38 cells in the indicated treatment groups (*n* = 6). (B) Survival rate of A549 cells in the indicated treatment groups (*n* = 6). (C, D) Experimental scheme (C) and tumor growth curves (D) of mice in inoculated with MC38 cells and treated with 18β‐GA + cisplatin or cisplatin alone (*n* = 6). (E–L) Concentration‐time profiles of 18β‐GA in plasma (E), cochlea (F), heart (G), liver (H), tumor (I), lung (J), kidney (K), and brain (L) (*n* = 6). **p < 0.01, ****p < 0.0001.

While 18β‐GA exhibits protective activity against cisplatin‐induced ototoxicity, its clinical applicability depends on preserving the antitumor function of cisplatin. To determine whether 18β‐GA interfered with the antitumor efficacy of cisplatin, we performed in vitro experiments using different cancer cell lines (MC38 and A549). Cancer cells were first pre‐treated with 18β‐GA at different concentrations (20 and 40 μM) for 1 h, followed by cisplatin (15 μM) treatment for 48 h. CCK‐8 assays showed that 18β‐GA did not impair cisplatin‐triggered tumor cell death compared with the cisplatin‐alone group. Notably, 40 μM 18β‐GA further potentiated cisplatin‐mediated tumor‐cell killing (Figure 8A,B). We next established a xenograft model by inoculating C57BL/6 mice with MC38 cells. When tumor volume reached 100 mm^3^, mice were treated with cisplatin (4 mg/kg, intraperitoneal injection once every 4 days) and 18β‐GA (50 mg/kg, intragastric gavage once daily) (Figure 8C). The results revealed that cisplatin monotherapy effectively reduced tumor volume, and the combined treatment (cisplatin + 18β‐GA) group also exhibited potent tumor‐suppressive effects (Figure 8D).

To gain mechanistic insight into the systemic exposure and cochlear distribution underlying the otoprotective property of 18β‐GA, pharmacokinetic profiling of 18β‐GA in different tissues was performed. The distribution concentration results of 18β‐GA were shown in all tested tissues at 0.5, 1, 2, 4, 8, 16, 24, and 48 h (Figure 8E–L). The pharmacokinetic parameters of 18β‐GA were calculated and shown in Table 1. After intragastric administration, plasma 18β‐GA concentrations rose rapidly with a *C*_max_ of 97.02 ± 41.83 ng/mL at 1 h, demonstrating rapid systemic absorption of 18β‐GA. The plasma *T*_1/2_ of 18β‐GA following oral gavage was 9.06 ± 0.78 h. It was found that 18β‐GA efficiently penetrated the blood‐labyrinth barrier and was stably retained in the cochlear tissue for a prolonged period (Figure 8F). In other tissues, 18β‐GA concentrations rapidly rise to peak levels within 0.5 or 1 h, and are nearly completely eliminated by 24 h (Figure 8G–L).

**TABLE 1:** Pharmacokinetic parameters of 18β‐GA after intragastric administration in different tissues (*n* = 6).

PK parameters	Serum	Cochlea	Heart	Liver	Tumor	Lung	Kidney	Brain
*T*_1/2_ (h)	9.06 ± 0.78	342.30 ± 401.79	8.91 ± 4.09	4.23 ± 0.96	6.96 ± 2.48	8.84 ± 6.41	5.92 ± 0.85	9.23 ± 3.56
*T*_max_ (h)	0.83 ± 0.26	11.42 ± 10.14	0.50 ± 0.00	1.00 ± 0.00	0.58 ± 0.20	0.83 ± 0.26	3.00 ± 1.55	1.50 ± 1.22
*C*_max_ (ng/mL or ng/g)	97.02 ± 41.83	78.98 ± 8.03	109.97 ± 51.94	2746.20 ± 1292.56	201.05 ± 105.25	385.61 ± 288.49	160.87 ± 32.26	17.14 ± 11.70
MRT (h)	10.15 ± 0.77	24.35 ± 1.13	13.80 ± 6.04	6.72 ± 1.19	10.17 ± 2.69	14.07 ± 8.27	9.52 ± 0.84	13.88 ± 3.85
AUC_0‐∞_ (h*ng/mL or h*ng/g)	816.38 ± 141.41	31,873.72 ± 36,657.89	1041.58 ± 409.46	14,746.87 ± 6788.07	1909.05 ± 925.16	3703.96 ± 1651.847	2424.76 ± 640.65	212.04 ± 95.76
V (mL/kg or g/kg)	835,740.02 ± 256,295.69	747,584.36 ± 85,433.05	781,450.40 ± 663,623.60	24,462.31 ± 12,483.79	282,517.34 ± 78,069.43	201,210.45 ± 138,615.50	181,945.73 ± 27,537.52	3,661,561.54 ± 1,581,464.00
CL (mL/h/kg or g/h/kg)	63,156.92 ± 13,395.45	3460.26 ± 2606.54	55,053.67 ± 23,095.93	4340.48 ± 2817.99	35,000.74 ± 25,209.19	16,586.93 ± 8942.251	22,069.74 ± 6736.63	282,438.51 ± 135,198.90

## 4 Discussion

In the present study, we investigated the effects of the herbal formula AC591, a standardized extract of HGWD, on CIHL. Our results suggest that oral administration of AC591 can attenuate cisplatin‐induced elevations in hearing thresholds and the loss of cochlear hair cells, neurofilaments, and ribbon synapses. RNA‐seq and immunostaining analyses demonstrated that AC591 primarily protected against CIHL through the inhibition of p53‐mediated ferroptosis. Furthermore, our LC‐HRMS experiments revealed that 18β‐GA was the major compound in AC591 responsible for hearing protection, confirming that 18β‐GA targeted p53 and exhibited anti‐ferroptosis effects in the inner ear.

Cisplatin, one of the most potent and widely used drugs for the treatment of various solid tumors, has been a cornerstone of chemotherapeutic regimens since 1978. Despite its extensive therapeutic benefits across multiple cancers, the clinical application of cisplatin remains constrained by its nephrotoxicity and ototoxicity [19]. While nephrotoxicity can be partially mitigated through enhanced saline hydration and the administration of mannitol diuretics, the ototoxic effects of cisplatin are irreversible, with an incidence ranging from 20% to 70% [20, 21, 22]. Cochlear hair cells, which are crucial for converting mechanical energy into electrical signals, are primarily affected following cisplatin exposure in the cochlear endolymph, subsequently leading to damage of SGNs [23, 24]. In this study, we found that pretreatment with AC591 effectively prevented cisplatin‐induced hair cell loss and damage in SGNs. The ribbon synapses between inner hair cells and SGNs are highly specialized structures that regulate the vesicular release of neurotransmitters for signal transmission [3, 25]. Notably, the damage or loss of afferent ribbon synapses caused by cisplatin treatment was restored following AC591 administration. Therefore, here we first provided a systematic demonstration of AC591 otoprotection with functional evidence, indicating that AC591 is a promising candidate for the treatment of CIHL.

The tumor suppressor p53, widely recognized as the “guardian of the genome,” plays a critical role in regulating multiple physiological functions, including DNA damage repair, cell cycle arrest, apoptosis, autophagy, aging, and ferroptosis [26, 27, 28, 29, 30, 31]. In response to cellular stress, p53 expression increases rapidly, contributing to the activation of downstream genes involved in the pathological processes associated with neurodegenerative diseases such as stroke, Alzheimer’s disease, and Parkinson’s disease [32, 33, 34]. A growing body of evidence suggests that p53 serves as a key regulator in the progression of hearing loss [35, 36, 37]. The activation of the p53 pathway is a major determinant in cisplatin‐induced ototoxicity, whereas genetic or pharmacological ablation of p53 can prevent the apoptosis of hair cells and protect hearing function [38]. Furthermore, it has been reported that p53 inhibition effectively reduces the loss of cochlear hair cells and SGNs in presbycusis [39]. In this study, we observed that the p53 signaling pathway was predominantly activated in the mouse cochlea following cisplatin injury, as demonstrated by RNA‐seq analysis and immunostaining. The up‐regulation of the p53 signaling pathway was suppressed by AC591 treatment, indicating that p53 may be a target protein of AC591 and that this signaling pathway plays a crucial role in the mechanism of action of AC591 for hearing protection.

Ferroptosis is a form of non‐apoptotic, programmed cell death characterized by the accumulation of peroxidized phospholipids or ferrous ions [40]. It has been reported to be associated with various physiological and pathological processes, including metabolic diseases, immunosuppression, ischemic injuries, and neurological disorders [41, 42, 43, 44]. Accumulating evidence suggests that p53 is closely related to the modulation of ferroptotic responses [31, 45, 46]. p53 can regulate both canonical and non‐canonical ferroptosis pathways through transcriptional and posttranslational mechanisms [18, 47]. Furthermore, it has been documented that ferroptosis is involved in CIHL, and the inhibition of ferroptosis mitigates cisplatin‐induced ototoxicity [7, 48]. Therefore, we evaluated the effects of AC591 on cisplatin‐induced ferroptosis in cochlear hair cells. The results from C11 BODIPY and FerroOrange staining demonstrated that cisplatin induces an increase in lipid peroxidation levels and the accumulation of ferrous ions in cochlear hair cells, which was reversed by AC591 treatment. Additionally, 4‐HNE and 3‐NT are biomarkers of lipid peroxidation and oxidative stress, with elevated expression of 4‐HNE and 3‐NT serving as hallmarks of ferroptosis [49, 50]. We observed that AC591 could suppress cisplatin‐induced overexpression of 4‐HNE and 3‐NT in hair cells, suggesting that AC591 may regulate cisplatin‐induced ferroptosis, potentially through the inhibition of the p53 signaling pathway.

Herbal formulas typically comprise a diverse array of chemical constituents that exhibit highly intertwined molecular mechanisms of action, which renders their complete mechanistic elucidation inherently challenging. In consideration of the complex components of AC591, we next aimed to identify the active compounds that exert significant protective effects on auditory function in both serum and the cochlea following the oral administration of AC591. Through LC‐HRMS analysis, we identified 13 AC591 compounds in serum and three in the cochlea. Notably, 18β‐GA was detected in both serum and cochlea, suggesting that it might be a key component of AC591 in preventing CIHL. 18β‐GA has been reported to exhibit various pharmacological activities, including anti‐inflammatory, antioxidant, antimicrobial, and neuroprotective effects [51, 52, 53, 54]. Moreover, it has been utilized in the treatment of skin disorders, rheumatoid arthritis, liver injury, and neurodegenerative diseases [55, 56, 57, 58], but its specific role in CIHL has never been reported. Our results showed that 18β‐GA could bind to p53 and exert anti‐ferroptosis effects, significantly protecting against CIHL, reinforcing its role as the major active component of AC591 involved in hearing protection.

While p53 modulation is closely implicated in cancer therapy, a published study has demonstrated that targeted inhibition of p53 does not compromise the antitumor efficacy of cisplatin [38]. The potential reasons may include: (1) Cochlear hair cells and spiral ganglion neurons are terminally differentiated cells in which cisplatin‐induced cytotoxicity is highly dependent on p53‐mediated ferroptosis and apoptotic pathways. In contrast, the anticancer effect of cisplatin is exerted through multiple mechanisms, including DNA cross‐link formation, immunoregulation and oxidative damage. (2) A large proportion of solid tumors harbor p53 mutations or dysregulated p53 signaling, such that partial modulation of p53 activity does not abolish the chemotherapeutic effect of cisplatin. To directly clarify whether p53‐modulating activity interferes with cisplatin‐based chemotherapy, we further verified using a xenograft model that 18β‐GA exerts otoprotective effects against CIHL without compromising the antitumor efficacy of cisplatin. Furthermore, pharmacokinetic analyses confirmed that 18β‐GA was stably retained in the cochlea for an extended duration to exert its therapeutic functions. While 18β‐GA alone cannot fully account for the protective mechanism of AC591 against cisplatin‐induced ototoxicity, the otoprotective effect of 18β‐GA, mediated by targeting p53 to modulate ferroptosis, is consistent with that of AC591, suggesting that 18β‐GA represents one of the key bioactive constituents underlying the auditory protective efficacy of AC591.

In summary, our study provides evidence that the herbal formula AC591, a standardized extract of HGWD, can prevent CIHL by inhibiting p53‐mediated ferroptosis, with 18β‐GA identified as the principal active constituent for hearing protection. As AC591 has already been evaluated in clinical trials for chemotherapy‐induced peripheral neuropathy, and 18β‐GA is widely used in clinical practice with a well‐established safety record, our work expands the clinical indication of these existing agents to CIHL, for which there is currently no approved pharmacological treatment. This ready translatability represents an important practical contribution to the field.

## Author Contributions

Yunhao Wu, Peng Cao, Xiaolong Fu, and Cheng Cheng designed the study. Yunhao Wu, Yu Tao, and Lin Wei carried out the experiments. Jiaxin Liu, Wen Jiang, and Weiwei Shi analyzed the data. Qiuping Liu performed part of the experiments. Yunhao Wu wrote the manuscript. Peng Cao, Xiaolong Fu, and Cheng Cheng reviewed and prepared the final version of the manuscript. All authors have seen and approved the final version of the manuscript being submitted. They warrant that the article is the authors’ original work, hasn’t received prior publication and isn’t under consideration for publication elsewhere.

## Conflicts of Interest

The authors declare no conflicts of interest.

## Supporting information

Supporting Information S1

## Data Availability

The datasets generated and/or analyzed during the current study are available from the corresponding author on reasonable request.
