# Supplementary material for: Herbal Formula AC591 Protects Against Cisplatin‐Induced Hearing Loss by Inhibiting p53‐Mediated Ferroptosis
Source: Smart Med. 2026 Sep 4;5(5):e70054. doi: 10.1002/smmd.70054 (PMC13573969; doi:10.1002/smmd.70054)
Supplement: Supplementary file 1 [file SMMD-5-e70054-s001.docx]

**Supplemental Materials**

**Supplemental Figures**

**Fig. S1** Effect of AC591 on cisplatin-induced cochlear hair cell loss and apoptosis of spiral ganglion neurons (SGNs). **A** Immunostaining of hair cells with anti-Myosin 7a in frozen cochlear sections. Scale bar, 20 μm. **B** Quantification of the number of outer hair cells in the apical, middle, and basal turns. n = 3. **C** TUNEL staining of the apoptotic SGNs in frozen cochlear sections. Scale bar, 25 μm. **D** Quantification of TUNEL positive SGNs in the different groups. n = 3, **P* < 0.05, ***P* < 0.01, ****P* < 0.001, *****P* < 0.0001.

**Fig. S2** Base Peak Chromatogram (BPC) and Total Ion Chromatography (TIC) of AC591 constituents in serum. **A** BPC of AC591 constituents in serum int negative-ion mode. **B** BPC of AC591 constituents in serum in positive-ion mode. **C** TIC of AC591 constituents in serum in negative-ion mode. **D** TIC of AC591 constituents in serum in positive-ion mode. Control: Blank serum without treatment. Treatment: Serum of mice treated with AC591. AC591: The original AC591 prescription.

**Fig. S3** Base Peak Chromatogram (BPC) and Total Ion Chromatography (TIC) of AC591 constituents in the cochlea. **A** BPC of AC591 constituents in the cochlea in negative-ion mode. **B** BPC of AC591 constituents in the cochlea in positive-ion mode. **C** TIC of AC591 constituents in the cochlea in negative-ion mode. **D** TIC of AC591 constituents in the cochlea in positive-ion mode. Control: Blank cochleae without treatment. Treatment: Cochleae from mice treated with AC591. AC591: The original AC591 prescription.
